# Predictors of physical activity and public safety perception regarding technology adoption for promoting physical activity in Jeddah, Saudi Arabia

**DOI:** 10.1016/j.pmedr.2024.102753

**Published:** 2024-05-10

**Authors:** Najim Z. Alshahrani

**Affiliations:** Department of Family and Community Medicine, Faculty of Medicine, University of Jeddah, Jeddah, Saudi Arabia

**Keywords:** Walking, High-intensity physical activity, Public health, Saudi Arabia

## Abstract

**Background:**

This study aims to identify the predictors of levels of physical activity among the residents of Jeddah, Saudi Arabia. Additionally, it seeks to understand the public's safety perception regarding the adoption of technology for promoting physical activity.

**Method:**

This is an analytical cross-sectional study using self-administered web-based survey. A stratified sampling technique was used to select participants from adult population in Jeddah and data collection took place from May 2023 to December 2023. Multivariable logistic regression models were applied to identify predictors of low-intensity (walking steps) and high-intensity physical activities.

**Result:**

A total of 462 participated in the study. Gender significantly influenced walking habits, with males more likely to walk than females (AOR = 2.37; 95 % CI: 1.55–3.62; P < 0.001). Smoking status was also a predictor, with smokers more inclined to walk compared to non-smokers (AOR = 2.15; 95 % CI: 1.20–3.82; P = 0.010). High-intensity physical activity strongly correlated with increased walking, especially among those active 3–7 days per week (AOR = 3.03; 95 % CI: 1.91–4.78; P < 0.001). Chronic diseases negatively impacted walking frequency (AOR = 0.57; 95 % CI: 0.34–0.95; P = 0.031). Furthermore, males were likelier to engage in high-intensity activities compared to females (AOR = 1.61; 95 % CI: 1.04–2.50; P = 0.033). Those walking ≥ 10,000 steps weekly were more likely to participate in high-intensity activities (AOR = 3.07; 95 % CI: 1.95–4.82; P < 0.001). Excellent self-rated health was associated with higher participation in such activities (AOR = 2.00; 95 % CI: 1.30–3.06; P = 0.002). Most respondents (84.9 %) felt safe on public walkways, and 78.1 % indicated safety perceptions influenced their walkway use. Technology use was divided, with about half (49.1 %) using apps for tracking physical activity. The majority (90.9 %) supported adding motivational features to the 'Sehhaty' app, and 73.6 % favored financial rewards for walking. However, only 45.5 % believed that public walkways are designed to encourage walking.

**Conclusion:**

The study highlights significant predictors of physical activity among general population in Jeddah. The study also revealed the impact of safety perceptions and technology use on physical activity, with strong support for motivational features in health apps. However, there is a need for improved design of public walkways to encourage walking.

## 1 Introduction

The World Health Organization (WHO) defines physical activity as “any bodily movement produced by skeletal muscles that results in energy expenditure” (WHO, 2024). Physical activity encompasses all forms of movement, not just during leisure time but also as part of a person's work and for transportation purposes (WHO, 2024). Both moderate- and vigorous-intensity physical activities are beneficial for health (Rey Lopez et al., 2020). Popular forms of physical activity include walking, cycling, wheeling, sports, active recreation, and play, which can be enjoyed by individuals of all skill levels and ages (Rey Lopez et al., 2020). The global issue of physical inactivity poses a significant public health challenge (Park et al., 2020). Approximately 27.5 % of adults and a staggering 81.1 % of adolescents worldwide do not meet the recommended levels of physical activity (WHO, 2024). This trend is especially pronounced in high-income countries, characterized by reduced physical exertion and increased sedentary behavior (Guthold et al., 2001). Recognizing this critical issue, the WHO urges countries around the world to develop and implement policies promoting physical activity (WHO, 2024). This is crucial, as regular physical activity plays a key role in preventing a variety of non-communicable diseases, including heart disease, obesity, diabetes, and some cancers (Anderson and Durstine, 2019). Moreover, individuals who are insufficiently active face a 20 % to 30 % higher risk of death compared to those who are sufficiently active (WHO, 2024, Haileamlak, 2019). Beyond its physical benefits, physical activity is essential for maintaining mental health and enhancing overall well-being (You et al., 2023d).

In the context of Saudi Arabia, the situation mirrors global trends but with distinct regional characteristics. Statistics reveal that the prevalence of diseases linked to physical inactivity, like heart disease and diabetes, is substantially high, with rates exceeding global averages by over 30 % (Al-Hazzaa, 2018). Additionally, obesity rates are notably elevated, and surveillance research indicates alarmingly low levels of physical activity among both men and women (Alsulami et al., 2023). This scenario underscores a pressing need to understand the factors influencing physical activity behavior among Saudi adults to inform effective health promotion strategies. Research in Saudi Arabia and neighboring Gulf countries has revealed a paucity of comprehensive studies focusing on the barriers to physical activity. Especially concerning are the low levels of activity among young people and females, which have been identified as priority areas for study (Al-Hazzaa, 2018). Although some research exists on specific population sectors, such as college students and primary care patients, there remains a significant gap in understanding these barriers across the general Saudi population (Osabi et al., 2023, Aljehani et al., 2022, Almaqhawi, 2021). Furthermore, with the socio-cultural landscape of Saudi Arabia undergoing rapid changes, particularly post launching of Vision 2030, there is an urgent need for contemporary research, especially concerning Saudi resident’s participation in physical activity.

The Saudi government has demonstrated strong support for promoting physical activity, particularly among women, with initiatives such as introducing sports in girls' schools and establishing women's sports competitions (Albujulaya and Stevinson, 2023). Additionally, the Ministry of Health has acknowledged the importance of walking (low-intensity physical activity) for public health by celebrating the National Day for Walking (You et al., 2023c). The ministry's objectives include promoting public health, fostering a culture of walking, increasing the number of walkers, and assisting those suffering from chronic diseases. In line with these goals, the ministry has announced the launch of a walking challenge (You et al., 2023c). Participants in this challenge are encouraged to set a daily goal of walking a minimum of 8,000 steps. To facilitate participation, individuals are encouraged to download and register on the “Sehhaty” app (Arab, 2024). Furthermore, in Riyadh, the Sports Boulevard, currently under construction, aims to encourage residents to embrace a healthier lifestyle by engaging in various sports like walking, cycling, and horse riding (Sports, 2024). The project aims to offer events and activities that would foster a vibrant cultural and recreational atmosphere. However, the effectiveness of investment in increasing physical activity requires further investigation in Saudi Arabia. Moreover, the influence of outdoor environments in promoting physical activity remains an under-explored area in Saudi Arabia. Despite the known health benefits of outdoor physical activity, including enhanced mental well-being and greater adherence, most research in this domain has predominantly been conducted outside the Middle Eastern context.

Saudi Vision 2030 has introduced a social-centric perspective, with a particular focus on enhancing the well-being of its citizens (Albujulaya and Stevinson, 2023). A key objective of this visionary plan is to encourage widespread participation in physical activities within the community, with an emphasis on individuals engaging in physical activity for at least 30 min per week. This initiative serves as a significant benchmark within the Quality-of-Life Program of the Vision Program. Notably, the emphasis on improving people's quality of life through such initiatives is a progressive concept that is gaining traction not only in developed nations but also in less developed countries. Given this background, there is a need for population-based studies which have been reported to be important in understanding physical activity (You et al., 2023c, You et al., 2024c). Therefore, this study aims to identify the predictors of low-intensity and high-intensity physical activity among the residents of Jeddah, Saudi Arabia. Additionally, the study seeks to understand the public's safety perception regarding the adoption of technology for facilitating physical activity. By focusing on these aspects, the study aims to contribute valuable insights into the facilitators and barriers of physical activity in an urban Saudi context, thereby informing policy and practice in health promotion and urban planning.

## 2 Methods

### 2.1 Study Design and Settings

This analytical cross-sectional study aimed to identify predictors of physical activity and to assess public safety perceptions regarding the adoption of technology for physical activity from May 2023 to December 2023. This design was chosen for its effectiveness in capturing a snapshot of behaviors and attitudes within the population at a single point in time (Kesmodel, 2018, Capili, 2021). The study period was selected to cover various seasonal changes and public holidays, which are likely to influence physical activity patterns and technology usage in outdoor settings. The study was conducted in Jeddah, a major city in Saudi Arabia known for its diverse population (Murad, 2014). The city's unique demographic and urban layout make it an ideal location for studying physical activity patterns and technology adoption in an urban Saudi population.

### 2.2 Sampling and Participants

A stratified sampling technique was employed in this study, initially selecting four renowned walking sites in Jeddah as primary strata. These sites were specifically chosen for their popularity and representation of different areas within the city, ensuring a diverse and comprehensive sample. Each stratum was chosen based on geographic and demographic criteria to enhance the representativeness and ensure that the findings could be generalized to the entire city. From these stratified locations, further sampling was conducted to select participants, thereby encompassing a wide array of Jeddah's adult population. A total of 444 participants were targeted, considering the city's diverse demographic profile and the expected prevalence of physical activity levels. This calculated sample size was crucial for an in-depth analysis of factors influencing physical activity and safety perceptions. The study welcomed participants aged 18 and above residing in Jeddah. It excluded those who refused participation or provided incomplete data. Calculations for the required sample size, initially set at 384 based on a 95 % confidence level and a 5 % margin of error, took into account an estimated physical activity engagement rate of 15 %-18 %. However, to account for potential non-responses or exclusions and to enhance the robustness of our findings, the total number of participants was eventually increased to 462. This methodical approach in employing stratified sampling from selected walking sites ensured a rich and representative dataset for our analysis.

### 2.3 Data Collection

Data were collected using a web-administered survey through Google Forms. This platform was chosen for its accessibility and ease of use, allowing participants to complete the questionnaire at their convenience. Before the questionnaire was uploaded to Google Forms, content validity was established by having it reviewed by two experts in survey research. Minor modifications were made to the survey accordingly. Notably, the questionnaire was crafted in Arabic to cater to the local population in Jeddah, ensuring that participants could understand and respond accurately, thereby enhancing the reliability of the data collected. The survey included a mix of structured questions and multiple-choice items designed to capture detailed information on physical activity levels and safety perceptions.

The questionaire was also used to collect data on various socio-demographic characteristics including age, gender, education level, marital status, employment status, income level, Body Mass Index (BMI) categories, smoking status, self-rated health quality, and the presence of chronic diseases (You et al., 2023d, You et al., 2023c, You et al., 2024c). The questionnaire included a specific question to assess low-intensity physical activity: “How many steps do you take per week?” This allowed us to categorize physical activity levels based on step counts (<10,000 steps per week indicating lower physical activity and ≥ 10,000 steps per week indicating higher activity). For high-intensity physical activity, I incorporated a question from the International Physical Activity Questionnaire (IPAQ) (Hagströmer et al., 2006): “During the last 7 days, on how many days did you do vigorous physical activities like heavy lifting, digging, aerobics, or fast bicycling?” Responses to this question were categorized into '0 to 2 days per week' and '3 to 7 days per week' to differentiate between lower and higher frequencies of high-intensity activities.

### 2.4 Statistical Analysis

Statistical analyses were conducted using Stata 18 software. Descriptive statistics were employed to summarize the socio-demographic characteristics of the study participants. Bivariate associations were determined using the Chi-Square test of independence for categorical variables. However, I chose the Wilcoxon rank-sum test to analyze bivariate associations for continuous variables due to their non-normal distribution, a decision supported by the test's suitability for comparing medians from independent samples when the assumptions of normality are not met. This choice ensures the robustness of our findings even in the presence of skewed data distributions. I considered potential predictors such as age, gender, education level, marital status, employment status, income level, Body Mass Index categories, smoking status, self-rated health quality, and the presence of chronic diseases. These factors were selected based on their relationship with levels of physical activity and general health outcomes. Only factors that were statistically significant at p < 0.05 were included in the multivariable logistic regression models. These models were applied to identify actual predictors of physical activities, categorized into low-intensity (e.g., walking steps) and high-intensity (measured by the IPAQ). The models reported the adjusted odds ratios, 95 % confidence intervals, and p-values. I also assessed public safety perceptions regarding the use of technology for physical activity, which was reported using descriptive statistics. Variance Inflation Factor analysis was performed after each regression model to detect multicollinearity, confirming its absence in our models. A p-value of less than 0.05 was considered statistically significant.

### 2.5 Ethical approval and considerations

This study adhered strictly to the ethical standards outlined in the Declaration of Helsinki. Ethical approval was granted by the Institutional Review Board of the Research Ethics Committee at the University of Jeddah, under the registration number HAP-02-J-094 and application number UJ-REC-217, with the official date of approval being January 16, 2023. This approval encompasses all research and data collection methods employed within the study, ensuring the protection of human subjects in accordance with institutional guidelines.

## 3 Results

A total of 462 participated in the study. Table 1 showed notable differences in socio-demographic characteristics between participants walking less than 10,000 steps per week (n = 213) and those walking 10,000 or more steps (n = 249). There was a significant difference in median age between the groups (P = 0.017), with older participants (median age 37) walking fewer than 10,000 steps, compared to a younger median age of 28 in the more active group. Gender distribution varied significantly (P < 0.001), with 35.2 % males in the less active and 62.7 % in the more active group. Marital status (P = 0.004) and income level (P = 0.047) also showed significant differences, with more singles and low-income individuals in the ≥ 10,000 steps group. Significant differences were observed in high-intensity physical activity levels and smoking status between groups (P < 0.001 for both), with more active smokers and high-intensity exercisers in the ≥ 10,000 steps group. Differences in self-rated health quality and chronic diseases were also significant (P = 0.016 and P = 0.013, respectively), with the more active group reporting better health but more chronic diseases.

**Table 1:** Descriptive statistics of the socio-demographic characteristics of a sample of adults in Jeddah by walking category, 2023.

Variable	< 10,000 steps per week (n = 213)	≥ 10,000 steps per week (n = 249)	P-Value
**Age, median (IQR), years**	37 (22–––48)	28 (22–42)	0.017*

**Gender, n (%)**			
Male	75 (35.2)	156 (62.7)	<0.001*
Female	138 (64.8)	93 (37.3)	

**Education, n (%)**			0.306
Higher education (Bachelor/Masters/PhD)	158 (74.2)	174 (69.9)	
Secondary or less	55 (25.8)	75 (30.1)	

**Marital status, n (%)**			0.004*
Single	92 (43.2)	141 (56.6)	
Married	121 (56.8)	108 (43.4)	

**Employment status, n (%)**			0.857
Unemployed	37 (17.4)	48 (19.3)	
Employed	99 (46.5)	119 (47.8)	
Retired	14 (6.6)	13 (5.2)	
Student	63 (29.6)	69 (27.7)	

**Income level, n (%)**			0.047*
Low income	42 (19.7)	67 (26.9)	
Middle income	109 (51.2)	100 (40.2)	
High income	62 (29.1)	82 (32.9)	

**BMI, n (%)**			0.284
Underweight	9 (4.2)	20 (8.0)	
Normal weight	77 (36.2)	97 (39.0)	
Overweight	80 (37.6)	84 (33.7)	
Obesity	47 (22.1)	48 (19.3)	

**High-intensity PA, n (%)**			<0.001*
0 to 2 days per week	175 (82.2)	141 (56.6)	
3 to 7 days per week	38 (17.8)	108 (43.4)	

**Smoker, n (%)**			<0.001*
Yes	22 (10.3)	59 (23.7)	
No	191 (89.7)	190 (76.3)	

**Self-rated health quality, n (%)**			0.016*
Good	139 (65.3)	135 (54.2)	
Excellent	74 (34.7)	114 (45.8)	

**Has chronic diseases, n (%)**			0.013*
Yes	55 (25.8)	41 (16.5)	
No	158 (74.2)	208 (83.5)	

*Statistically significant P-value < 0.05.

Table 2, which presents the outcomes of our multivariable logistic regression analysis, highlights several significant predictors of walking behavior among the study participants. Age was not a significant factor in walking habits (AOR = 0.99; 95 % CI: 0.98–1.02; P = 0.861), while gender significantly influenced walking, with males more likely to walk than females (AOR = 2.37; 95 % CI: 1.55–3.62; P < 0.001). Income levels did not predict walking behavior, nor did marital status. Smoking status was a significant predictor, with smokers more likely to walk (AOR = 2.15; 95 % CI: 1.20–3.82; P = 0.010). High-intensity physical activity strongly influenced walking likelihood, particularly among those active 3–7 days per week (AOR = 3.03; 95 % CI: 1.91–4.78; P < 0.001). Chronic diseases negatively affected walking likelihood (AOR = 0.57; 95 % CI: 0.34–0.95; P = 0.031), highlighting key factors like gender, smoking status, and physical activity as significant influences on walking behavior.

**Table 2:** Predictors of walking among a sample of adults in Jeddah, 2023.

Variable	AOR^a^ (95 % CI)	P-Value
**Age**	0.99 (0.98–1.02)	0.861

**Gender**		
Male	2.37 (1.55–3.62)	<0.001*
Female	Ref	

**Income level**		
Middle income	0.69 (0.41–1.17)	0.166
High income	0.93 (0.53–1.62)	0.795
Low income	Ref	

**Marital Status**		
Married	0.85 (0.48–1.5)	0.579
Single	Ref	

**Smoker**		
Yes	2.15 (1.20–3.82)	0.010*
No	Ref	

**High-intensity physical activity**		
3 to 7 days per week	3.03 (1.91–4.78)	<0.001*
0 to 2 days per week	Ref	

**Self-rated health quality**		
Excellent	1.24 (0.82–1.89)	0.298
Good	Ref	

**Has chronic diseases**		
Yes	0.57 (0.34–0.95)	0.031*
No	Ref	

^a^The odds ratios have been adjusted for all other variables in the model.

*Statistically significant P-value < 0.05.

In Table 3, the socio-demographic characteristics of the study participants were explored, categorized by their engagement in high-intensity physical activity. Participants were split into two groups based on high-intensity physical activity: 0 to 2 days per week (n = 316) and 3 to 7 days per week (n = 146). Analysis showed no significant age difference between the groups (P = 0.438), though the more active group was younger. Gender distribution differed significantly (P < 0.001), with more males in the 3 to 7 days group, indicating a gender-activity level correlation. Education, marital status, employment status, and BMI showed no significant differences. Income level hinted at a potential influence on physical activity (P = 0.053). Walking habits differed significantly (P < 0.001), with those more active in high-intensity exercise also walking over 10,000 steps per week. Smoking status differences were not statistically significant (P = 0.247). Self-rated health quality varied significantly (P < 0.001), with the more active group reporting better health. No significant difference was seen in chronic diseases (P = 0.410). These findings suggest gender, walking habits, and self-rated health as key factors associated with high-intensity physical activity frequency.

**Table 3:** Descriptive statistics of socio-demographic characteristics of the study participants by high-intensity physical activity category, 2023.

Variable	0 to 2 days per week (n = 316)	3 to 7 days per week (n = 146)	P-Value
**Age, median (IQR), years**	33 (22–46)	29 (22–42)	0.438

**Gender, n (%)**			
Male	138 (43.7)	93 (63.7)	<0.001*
Female	178 (56.3)	53 (36.3)	

**Education, n (%)**			0.516
Higher education (Bachelor/Masters/PhD)	230 (72.8)	102 (69.9)	
Secondary or less	86 (27.2)	44 (30.1)	

**Marital status, n (%)**			0.382
Single	155 (49.0)	78 (53.4)	
Married	161 (51.0)	68 (46.6)	

**Employment status, n (%)**			0.726
Unemployed	57 (18.0)	28 (19.2)	
Employed	147 (46.5)	71 (48.6)	
Retired	17 (5.4)	10 (6.9)	
Student	95 (30.1)	37 (25.3)	

**Income level, n (%)**			0.053
Low income	69 (21.8)	40 (27.4)	
Middle income	155 (49.1)	54 (36.9)	
High income	92 (29.1)	52 (35.7)	

**BMI, n (%)**			0.529
Underweight	20 (6.2)	9 (6.2)	
Normal weight	113 (35.8)	61 (41.8)	
Overweight	113 (35.8)	51 (34.9)	
Obesity	70 (22.2)	25 (17.1)	

**Walking category, n (%)**			<0.001*
< 10,000 steps per week	175 (55.4)	38 (26.0)	
≥ 10,000 steps per week	141 (44.6)	108 (74.0)	

**Smoker, n (%)**			0.247
Yes	51 (16.1)	30 (20.6)	
No	265 (83.9)	116 (79.4)	

**Self-rated health quality, n (%)**			<0.001*
Good	207 (65.5)	67 (45.9)	
Excellent	109 (34.5)	79 (54.1)	

**Has chronic diseases, n (%)**			0.410
Yes	69 (21.8)	27 (18.5)	
No	247 (78.2)	119 (81.5)	

*Statistically significant P-value < 0.05.

In Table 4, the findings from the multivariable logistic regression analysis are presented, aimed at identifying the predictors of high-intensity physical activity among the study participants. The analysis provided insights into factors influencing high-intensity physical activity. Age was not significant (AOR = 1.00; 95 % CI: 0.99–1.02, P = 0.389), while gender showed a notable impact, with males more likely than females to engage in high-intensity activities (AOR = 1.61; 95 % CI: 1.04–2.50, P = 0.033). Walking habits were a strong predictor; those walking ≥ 10,000 steps per week were more likely to engage in high-intensity activities (AOR = 3.07; 95 % CI: 1.95–4.82, P < 0.001). Self-rated health quality also significantly influenced activity levels, with those rating their health as excellent more likely to participate in high-intensity activities compared to those rating it as good (AOR = 2.00; 95 % CI: 1.30–3.06, P = 0.002). These results highlight the roles of gender, walking habits, and self-perceived health in high-intensity physical activity engagement.

**Table 4:** Predictors of high-intensity physical activity in a sample of adults in Jeddah, 2023.

**Variable**	**AOR^a^ (95 % CI)**	**P-Value**
**Age**	1.00 (0.99–1.02)	0.389

**Gender**		
Male	1.61 (1.04–2.50)	0.033*
Female	Ref	

**Walking category**		
≥ 10,000 steps per week	3.07 (1.95–4.82)	<0.001*
< 10,000 steps per week	Ref	

**Self-rated health quality**		
Excellent	2.00 (1.30–3.06)	0.002*
Good	Ref	

^a^The odds ratios have been adjusted for all other variables in the model.

*Statistically significant P-value < 0.05.

The survey investigated the public’s perception of safety while walking and their attitudes towards technology use in promoting physical activity. The results, as shown in Table 5, provide valuable insights into these aspects. A majority (84.9 %, n = 392) felt safe walking on public walkways, while 15.1 % (n = 70) did not. Safety perception significantly influenced 78.1 % (n = 361) of participants' use of walkways, emphasizing safety's role in encouraging walking. Technology use was evenly divided: 49.1 % (n = 227) used activity-tracking apps, while 50.9 % (n = 235) did not, suggesting growth potential in tech adoption for physical activity. A vast majority (90.9 %, n = 420) supported adding features to the 'Sehhaty' app to encourage walking, showing a positive attitude towards health app enhancements. Furthermore, 73.6 % (n = 340) favored financial rewards based on steps and distance, indicating strong support for incentivizing physical activity. However, only 45.5 % (n = 210) believed public walkways were well-prepared for encouraging walking, while 54.5 % (n = 252) disagreed, pointing to a need for infrastructure improvements. These findings indicate a general sense of safety and positive technology outlook but highlight areas for improvement in infrastructure and technology adoption, suggesting app enhancements and financial incentives as potential strategies to boost physical activity.

**Table 5:** Safety perception of the public regarding walking and technology adoption for physical activity among a sample of adults in Jeddah, 2023.

**Perception questions**	**n (%)**
**Do you feel safe while walking on the public walkway?**	
Yes	392 (84.9)
No	70 (15.1)
**Does your use of the public walkway depend on your perception of safety in the place?**	
Yes	361 (78.1)
No	101 (21.9)
**Do you use apps to track physical activity while walking?**	
Yes	227 (49.1)
No	235 (50.9)
**Do you support adding a feature to encourage walking in the (Sehhaty) app?**	
Yes	420 (90.9)
No	42 (9.1)
**Do you think that the public walkway is prepared in a way that encourages people to walk?**	
Yes	210 (45.5)
No	252 (54.5)
**Do you support financial rewards based on the number of steps and kilometers traveled?**	
Yes	340 (73.6)
No	122 (26.4)

## 4 Discussion

This study aimed to identify predictors of both low-intensity and high-intensity physical activity, as well as to understand public safety perceptions and technology adoption for physical activity in Jeddah, Saudi Arabia. The present study findings indicate that gender significantly influences walking behavior, aligning with global trends where men often report higher levels of physical activity and daily step counts than women. For instance, a study conducted in the Australia (van Uffelen et al., 2017), United States (Li et al., 2017) and previous study in Riyadh (Bashatah et al., 2023) revealed similar gender disparities in physical activity levels. In addition, some studies have also revealed a constrast result where females have higher daily step counts than males (Cho et al., 2013). However, in contrast to some reports from European countries where age is a determinant (Martins et al., 2023, Jylhä et al., 2001, LaRoche et al., 2018), this study found that age did not significantly impact walking habits in Jeddah. This different could be attributed to cultural and environmental differences, such as societal norms and urban design, which influence physical activity across different age groups.

The significant association between gender and high-intensity physical activity in this study is consistent with findings from other regions, including the Middle East and Western countries (Zimmermann-Sloutskis et al., 2010, Azevedo et al., 2007, Reading and LaRose, 2022). Men are generally more inclined towards high-intensity activities, possibly due to cultural perceptions and opportunities available. Interestingly, our results also show that those who walk more are likely to engage in high-intensity activities, suggesting a positive correlation between different forms of physical activity. The current study found that smokers were more likely to engage in walking activities, an intriguing result considering the general health risks associated with smoking. This finding contrasts with some local studies in Saudi Arabia, which typically show lower physical activity levels among smokers (Abdulrashid et al., 2023). For instance, a study conducted in Riyadh reported a negative correlation between smoking and physical activity (Alobaid et al., 2023). This discrepancy might be attributed to varying lifestyle patterns or stress-coping mechanisms where individuals in urban settings like Jeddah use walking as a form of stress relief, including those who smoke.

The strong correlation between high-intensity physical activity and walking aligns with findings from other Saudi Arabian cities, where active individuals often engage in multiple forms of physical exercise (Alhammad et al., 2023, Karmakar et al., 2023). This reflects a broader trend observed in the region, where a lifestyle that includes one form of physical activity often correlates with other types. It suggests an overall active lifestyle preference among certain segments of the population, contrary to the prevailing sedentary lifestyle trend in the region (Al-Hazzaa, 2018). The results indicate that participants with chronic diseases were less likely to engage in walking. This is consistent with other studies in Saudi Arabia and internationally that have identified chronic illnesses as a barrier to physical activity (Booth et al., 2012, Alqahtani et al., 2021, Munawir Alhejely et al., 2023). For example, a systematic review from Saudi Arabia found that individuals with chronic conditions, like diabetes and hypertension, often had lower physical activity levels, possibly due to physical limitations or fear of exacerbating their condition (Pastor-Cisneros et al., 2021). This underscores the need for tailored physical activity programs that accommodate and encourage safe exercise for individuals with chronic diseases (You et al., 2023d, You et al., 2023c).

Participants who perceived their health as excellent were more likely to engage in high-intensity activities. This aligns with studies from various parts of the world, indicating that self-perceived health status is a strong motivator for engaging in physical activity (Buchmann et al., 2023, Lenhart et al., 2017). It underscores the psychological aspect of physical activity, where personal health perception significantly influences one’s lifestyle choices. The survey results show a strong sense of safety among the majority of participants when walking in public areas, a crucial factor for outdoor physical activity. This finding is in line with international studies emphasizing the importance of perceived safety in public spaces for promoting physical activity (Juul and Nordbø, 2023, Liu et al., 2023). Furthermore, the divided response towards the use of technology for tracking physical activity suggests an opportunity for increased adoption, a trend similarly observed in studies from other countries (Wang et al., 2019). The current study findings regarding the public's perception of walkways not being conducive to walking highlight a significant area for urban planning and development. This is a common issue in many urban centers globally, where the design of public spaces does not always encourage physical activity (Mitchell et al., 2020). The positive response to financial incentives for physical activity aligns with successful interventions in other countries, emphasizing the potential of such strategies in increasing activity levels (You et al., 2023d, You et al., 2024b).

Socio-economic status may have influenced our findings, as individuals from higher socio-economic backgrounds are potentially more likely to have access to technology for physical activity. Additionally, urban infrastructure such as the availability of parks and recreational areas could significantly affect physical activity levels across different regions of Jeddah. Cultural factors, including societal norms and attitudes towards physical activity, particularly among different genders, also play a crucial role in shaping physical activity patterns and technology adoption. Additionally, this emphasizes the importance of population-based studies in topics like this (Adebisi et al., 2024, Aleid et al., 2024, Alshahrani et al., 2021, Hagströmer et al., 2006, You et al., 2023a, You et al., 2023b, You et al., 2024a, Martínez-Mesa et al., 2016).

Overall, the findings of this study have several important implications for public health policy and intervention strategies in Jeddah. Given the significant gender differences in physical activity, targeted interventions that account for cultural norms and gender-specific preferences could enhance engagement in both walking and high-intensity activities. The strong correlation between self-perceived health and participation in high-intensity activities suggests that health awareness campaigns could motivate more residents to become active, emphasizing the benefits of physical activity on overall well-being. Additionally, considering the mixed responses towards technology use for tracking physical activity, there is a clear opportunity to increase adoption by improving accessibility and promoting the benefits of tech-based fitness tools (Tong et al., 2024, Adebisi et al., 2021). The perception of safety and inadequate walkway conditions also highlights the need for urban planning improvements to create more inviting and conducive public spaces for physical activity. Finally, the positive response to financial incentives for physical activity indicates that such strategies could be effective in encouraging a more active lifestyle among Jeddah residents. These recommendations could help refine current public health strategies and contribute to the broader goal of improving community health through increased physical activity.

The current study offers a comprehensive analysis of physical activity in Jeddah, exploring factors like walking habits, high-intensity physical activity, smoking status, and self-perceived health. The use of a large sample size enhances the statistical power and generalizability of our findings within the urban context of Jeddah. The inclusion of a diverse range of socio-demographic variables allows for a multifaceted understanding of physical activity behaviors. Furthermore, the employment of multivariable logistic regression analysis enables the identification of independent predictors of physical activity, providing valuable insights for targeted interventions. However, this study has limitations. The study design restricts the ability to establish causality between the observed factors and physical activity levels which is a characteristic of cross-sectional studies (Aleid et al., 2024). The use of convenience sampling may lead to selection bias, limiting the generalizability of its findings to the broader Saudi population. Additionally, the reliance on self-reported data for physical activity and health status could introduce response bias. The lack of objective measures of physical activity (e.g., accelerometers) might affect the accuracy of reported activity levels. Future research should focus on longitudinal designs to establish causal relationships between the observed variables and physical activity. Expanding the study to include other cities in Saudi Arabia would enhance the generalizability of the findings. Incorporating objective measures of physical activity alongside self-reported data would improve the accuracy and reliability of the results. Research should also explore the underlying reasons for the unique findings in this study, such as the increased likelihood of walking among smokers. Finally, interventions based on these findings should be developed and tested for their effectiveness in promoting physical activity in similar urban settings.

## 5 Conclusion

This study provides valuable insights into the predictors of physical activity, highlighting significant roles of gender, smoking status, chronic diseases, and self-perceived health. Despite its limitations, including a cross-sectional design and reliance on self-reported data, the study's comprehensive approach offers a nuanced understanding of physical activity behaviors in an urban setting. The study findings emphasize the need for targeted public health strategies, considering the unique socio-demographic and lifestyle factors identified. The study underscores the potential of using technology and improving public infrastructure to promote physical activity, as reflected in the respondents' attitudes towards safety and technology use. Future research should extend these findings through longitudinal studies and broader geographic sampling to deepen the understanding and applicability of these results. Ultimately, this study contributes significantly to the growing body of knowledge on physical activity patterns in the Middle East, guiding policy, and intervention design for healthier, more active communities.

## Source of funding

This research received no external funding.

**Ethical approval**.

The study was conducted in accordance with the Declaration of Helsinki and approved by the Institutional Review Board of Research Ethics Committee at University of Jeddah (Registration number HAP-02-J-094, application number: UJ-REC-217 and date of approval 16/01/2023).

## CRediT authorship contribution statement

**Najim Z. Alshahrani:** Writing – review & editing, Writing – original draft, Visualization, Validation, Supervision, Software, Resources, Project administration, Methodology, Investigation, Funding acquisition, Formal analysis, Data curation, Conceptualization.

## Declaration of competing interest

The author declares that he has no known competing financial interests or personal relationships that could have appeared to influence the work reported in this paper.

## Data Availability

Data will be made available on request.
